# The Experimental Development of Emulsions Enriched and Stabilized by Recovering Matter from *Spirulina* Biomass: Valorization of Residue into a Sustainable Protein Source

**DOI:** 10.3390/molecules28176179

**Published:** 2023-08-22

**Authors:** Anna Rafaela Cavalcante Braga, Maria Cristiana Nunes, Anabela Raymundo

**Affiliations:** 1 Department of Chemical Engineering, Campus Diadema, Federal University of São Paulo (UNIFESP), Diadema 09972-270, Brazil; anna.braga@unifesp.br; 2 Department of Biosciences, Federal University of São Paulo (UNIFESP), Silva Jardim Street 136, Vila Mathias, Santos 11015-020, Brazil; 3 LEAF-Linking Landscape, Environment, Agriculture and Food Research Center, Associate Laboratory TERRA, Instituto Superior de Agronomia, Universidade de Lisboa, Tapada da Ajuda, 1349-017 Lisboa, Portugal; cristiananunes@isa.ulisboa.pt

**Keywords:** residual biomass, antioxidant activity, technological ingredient, food industry, vegan emulsion

## Abstract

*Spirulina* consists of a cluster of green-colored cyanobacteria; it is commonly consumed as a food or food supplement rich in bioactive compounds with antioxidant activity, predominantly C-phycocyanin (C-PC), which is related to anti-inflammatory action and anticancer potential when consumed frequently. After C-PC extraction, the *Spirulina* residual biomass (RB) is rich in proteins and fatty acids with the potential for developing food products, which is interesting from the circular economy perspective. The present work aimed to develop a vegan oil-in-water emulsion containing different contents of *Spirulina* RB, obtaining a product aligned with current food trends. Emulsions with 3.0% (*w*/*w*) of proteins were prepared with different chickpea and *Spirulina* RB ratios. Emulsifying properties were evaluated regarding texture and rheological properties, color, antioxidant activity, and droplet size distribution. The results showed that it was possible to formulate stable protein-rich emulsions using recovering matter rich in protein from *Spirulina* as an innovative food ingredient. All the concentrations used of the RB promoted the formulation of emulsions presenting interesting rheological parameters compared with a more traditional protein source such as chickpea. The emulsions were also a source of antioxidant compounds and maintained the color for at least 30 days after production.

## 1. Introduction

A recent communication from the European Commission [1] discussed actions to promote the algae sector—expected to grow 8.7% by 2025 and to reach € 9 billion—in Europe for healthier diets, lower CO_2_ emissions, and water reuse. It also included cyanobacteria *Spirulina* (*Arthrospira platensis*) as a sustainable pathway [2,3,4,5]. Accordingly, multiple-partner efforts must be followed to improve food systems resilience [1,6,7] for population growth and mitigating deforestation. Healthier and sustainable food practices that respect the individual and the collective must contribute to addressing the current highly complex problem of obesity, malnutrition, and climate change [8].

The sustainability of *Spirulina* stands on an environmental, economic, and social basis. The cultivation process using wastewater and photobioreactors [9,10] gained environmental notoriety since it does not depend on soil, as agriculture usually does. In this sense, semiarid climatic conditions and uncommon cultivable soils could fit its production, also generating wealth and employment. The *Spirulina* biomass contains 57.5% protein, 23.9% carbohydrates, and 7.72% fatty acids [2,11,12,13,14,15,16,17,18]. Comparatively, the extensively consumed soy flour has 38.6% protein. Thus, *Spirulina* is an attractive source of food security as it is a new possibility for nourishment.

Several researchers have reported the great potential of *Spirulina* and its ingredients in the last decades [19,20,21,22,23,24,25]. A work presented by Uribe-Wandurraga et al. [26] about the addition of three microalgae biomass, including *Spirulina*, in low-fat oil-in-water emulsions made for food products application showed for each textural factor, a slight difference between control and *Spirulina* low-fat emulsions. These results are evidence of the influence of distinctive features and composition of microalgae as functional ingredients, showing the importance of developing studies as the present work to improve the application of these sustainable resources. The authors have also highlighted the microalgae protein isolate nanoparticle as an innovative way to stabilize emulsions [19,27,28,29].

Bearing in mind that after the C-PC extraction, all the other components are in the remaining material, the whole biomass (WB) can be used to create protein-rich and plant-based food products, and it is worth mentioning that after the C-PC’s extraction, the *Spirulina* residual biomass (RB) preserve their nutritional appeal been able to supplement foodstuff instead of being characterized as waste, and give them both functionality and naturally greenish color [30]. RB is underestimated—a gap that must be overcome to run the circular economy—and it became an aspiration for the present study.

This work focuses on the array for going forward the circular economy based on *Spirulina* WB and RB after extracting the natural blue colorant known as C-phycocyanin (C-PC) for healthier diets and innovation in the Food Industry [31,32,33]. Consumers are demanding to stay healthy, not only individually but alongside environmental preservation of fauna and flora, constituting the One Health concept [31], and vegetarianism and veganism are growing [34,35].

Considering that, the present work aimed to develop a vegan emulsion containing different contents of *Spirulina* residual biomass, acquiring food goods that support recent trends.

## 2. Results

### 2.1. Texture Analysis Results

**Figure 1:**
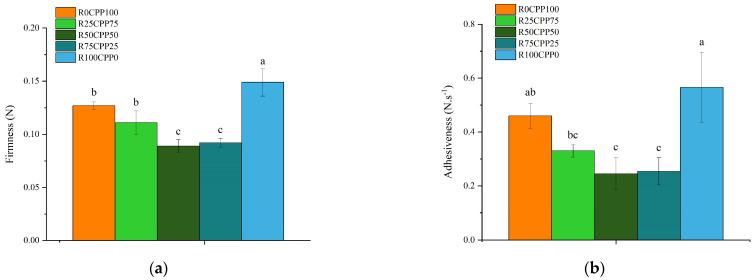
Firmness (N) (**a**) and adhesiveness (N.s^−1^) (**b**) of emulsions with 3% (*w*/*w*) of different combinations of proteins (chickpea protein and *Spirulina* residual biomass—RB). R0CPP100 (100% chickpea), R25CPP75 (25% RB and 75% chickpea), R50CPP50 (50% RB and 50% chickpea), R75CPP25 (75% RB and 25% chickpea) and R100CPP0 (100% RB without chickpea). According to the Tukey test, different letters indicate significantly different results (*p* < 0.05). The vertical bars indicate the standard deviation.

The results of texture parameters of the emulsions, firmness (Figure 1a), and adhesiveness (Figure 1b) seem to be dependent on the ratio of RB/CPP.

The formulation without RB (R0CPP100) was considered the standard or control (*p* < 0.05). R0CPP100 and R25CPP75 showed similar values considering both texture parameters, while when the RB percentage increased (R50CPP50 and R75CPP25), firmness and adhesiveness decreased. However, the emulsion composed only of RB as a protein source (R100CPP0) showed the highest values of both firmness and adhesiveness.

### 2.2. Rheology Results

**Figure 2:**
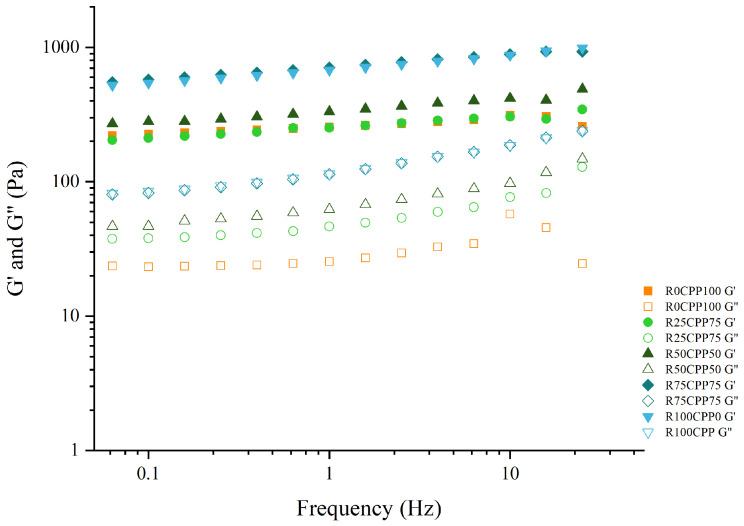
Mechanical spectra of emulsions with 3% (*w*/*w*) of different combinations of proteins (chickpea protein and *Spirulina* residual biomass—RB). R0CPP100 (100% chickpea), R25CPP75 (25% RB and 75% chickpea), R50CPP50 (50% RB and 50% chickpea), R75CPP25 (75% RB and 25% chickpea) and R100CPP0 (100% RB without chickpea). G′ corresponds to the elastic modulus, and G″ corresponds to the viscous modulus.

Figure 2 shows the mechanical spectra of formulated emulsions, i.e., the variation of G′ (elastic modulus) and G″ (viscous modulus) with the oscillation frequency in the viscoelastic linear zone. The shape of the curves is similar for all the emulsions, with G′ being higher than G″, indicating a predominantly elastic behavior. In addition, all the mechanical spectra show a similar slope and pattern. The emulsion’s rheological behavior is essential to understand the features of panorama [36,37,38,39,40,41,42], particularly considering food development.

**Figure 3:**
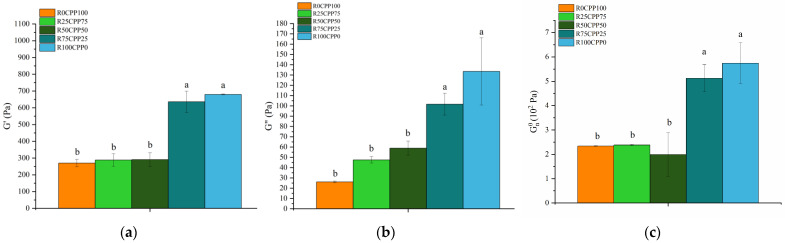
Elastic modulus G′ (**a**) and viscous modulus G″ (**b**) at 1 Hz, and plateau module G^0^_N_ (**c**) of the emulsions with 3% (*w*/*w*) of different combinations of proteins (chickpea protein and *Spirulina* residual biomass—RB). R0CPP100 (100% chickpea), R25CPP75 (25% RB and 75% chickpea), R50CPP50 (50% RB and 50% chickpea), R75CPP25 (75% RB and 25% chickpea) and R100CPP0 (100% RB without chickpea). According to the Tukey test, different letters indicate significantly different results (*p* < 0.05). The vertical bars indicate the standard deviation.

Figure 3 presents the elastic modulus G′, and viscous modulus G″ at 1 Hz, and plateau module (G^0^_N_) of the emulsion with 3% (*w*/*w*) of different combinations of proteins (chickpea protein and *Spirulina* residue biomass).

**Figure 4:**
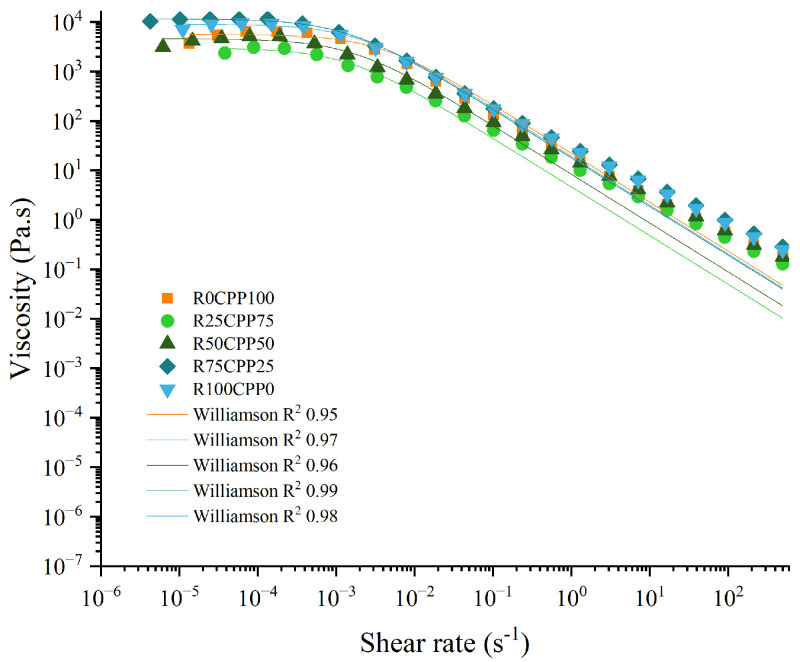
Flow curves of emulsions with 3% (*w*/*w*) of different combinations of proteins (chickpea protein and *Spirulina* residual biomass—RB). R0CPP100 (100% chickpea), R25CPP75 (25% RB and 75% chickpea), R50CPP50 (50% RB and 50% chickpea), R75CPP25 (75% RB and 25% chickpea) and R100CPP0 (100% RB without chickpea). Lines represent Williamson’s model adjustment.

Figure 4 illustrates the flow behavior of the emulsion with different proportions of chickpea protein and *Spirulina* residue. By analyzing Figure 4, all the emulsions showed the same flow trend, exhibiting a shear-thinning behavior typical of this type of protein-stabilized emulsions. For each emulsion formulation, Williamson’s parameters are presented in Table 1.

**Figure 5:**
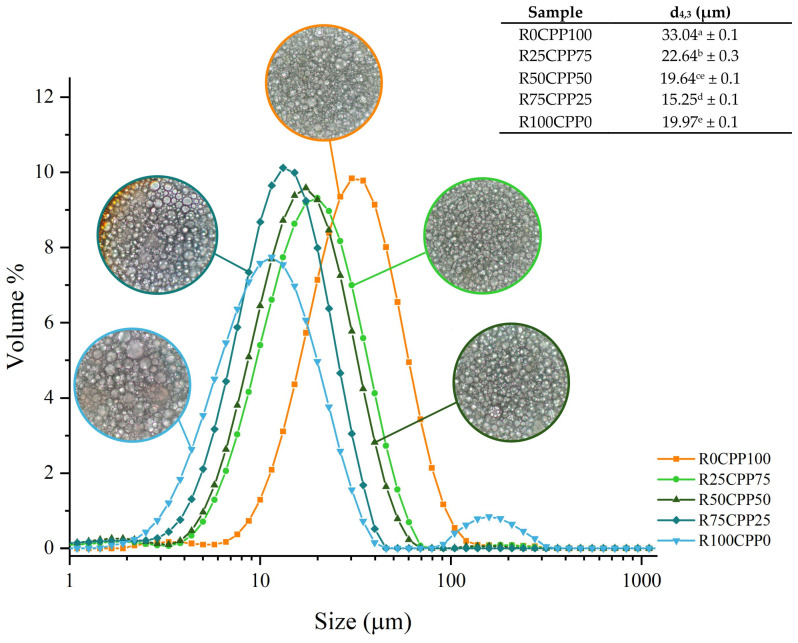
Determination of droplet size distribution and microscopy of the emulsions with 3% (*w*/*w*) of different combinations of proteins (chickpea protein and *Spirulina* residual biomass -RB) and De Brouckere diameter (d4,3) obtained from the droplet size distribution analysis. R0CPP100 (100% chickpea), R25CPP75 (25% RB and 75% chickpea), R50CPP50 (50% RB and 50% chickpea), R75CPP25 (75% RB and 25% chickpea) and R100CPP0 (100% RB without chickpea). According to the Tukey test, different letters (a, b, c, d and e) indicate significantly different results (*p* < 0.05).

Figure 5 shows the droplet size distribution (DSD) measurement results for the different emulsions formulated using a laser diffraction method and the appearance of the respective droplets by microscopy. Overall, the emulsions presented volumetric (d_4,3_) diameters ranging from 15.25 to 33.04 µm, values that are in the range of 100 nm to 100 µm for a conventional emulsion [43,44], also the smaller droplet sizes are related to more stable emulsions. The emulsions containing RB presented smaller droplet sizes than those prepared only with chickpea protein (R0CPP100), highlighting the R75CPP25, which showed the smallest values, d_4,3,_ of 15.25 µm, on average. Consequently, the RB may be more effective than chickpea in reducing the oil droplet size owing to the concentration difference of emulsifying components due to moisture content [45].

### 2.3. Color Parameters

Table 2 presents the results for color parameters of emulsions formulated at initial and final times (after 30 days) for brightness (L*), amount of green or red (a*), amount of blue or yellow (b*), and color (Hue angle). None of the formulations showed statistical differences between t = 0 and t = 30 d for L* a* b* and Hue angle.

In the formulation without the RB addition (R0CPP100), b* values were positive, and the Hue angle was 93.7° ± 0.23 and 92.96° ± 0.03 on initial time and final time, respectively, indicating slightly yellowish coloration, as expected due to the chickpea used to develop the emulsion. All the formulations showed negative a* values (Table 2), detecting the green colour in these samples. The formulation R0CPP100 showed significantly lower values, as expected, due to the RB being the source of the green colour.

### 2.4. Antioxidant Activity

Besides the maintenance of color presented as results, all the samples showed antioxidant activity that was determined using two methods (Table 3), as already mentioned, and the behavior of the results is very similar for both methods used.

Considering DPPH and FRAP analysis, the antioxidant activity ranged from 15.4 ± 0.16 to 43.0 ± 1.5 and from 169.38 ± 5.75 to 343.91 ± 21.12, respectively. The increment in the percentage of RB as a protein source in the emulsions led to higher antioxidant activities, in general. A previous work of our research group [30] showed that the RB partially preserved the antioxidant activity from the *Spirulina*, showing for the first time the potential of the residue as a food ingredient.

## 3. Discussion

Since microalgae present up to 70% protein accumulation in their dry matter, the fact that they grow quicker than terrestrial plants and reach better protein productivities per area than other crops such as soybean, legumes, or wheat, they might be viewed as a future sustainable protein source [29]. Considering the advances in the circular economy based on *Spirulina* WB and RB after extracting the C-PC for healthier diets and innovation in Food Industry, the present work shows an essential step in this direction. The results indicated that the interaction between CPP and RB, in specific percentages, changes the emulsions’ texture. At the same time, the outcomes in the sample containing only RB as a protein source are the opposite—firmness and adhesiveness increase, compared with the control sample.

An antagonistic effect among chickpea vegetable proteins and RB can be a hypothetical explanation for the fact that these proteins have a better ability to form emulsions when used separately rather than in mixtures. This behavior should result from a competition between the proteins for the interface, which manifests itself in a negative effect on the texture of the emulsions. The chickpea protein role in reinforcing the network structure of gels was highlighted by Ozcan et al. [45]. Reviewing the results presented in the literature regarding proteins from microalgae for the stabilization of fluid interfaces, emulsions, and foams, Bertsch et al. [46] affirm that microalgae proteins, including *Spirulina’s*, adsorb at fluid interfaces at comparable kinetics and surface tension reduction as whey protein isolate, one of the most common stabilizers in industry. Considering the emulsions produced in the present work, during storage, we observed that they remained visually stable for up to seventeen days. Still, the stability was not formally measured using a method since we present a more horizontal study. Further work will verticalize this baseline, producing a vegan mayonnaise and studying the formulation in a more panoramic way.

As reported in the literature, the interactions between different types of emulsifiers determine the interfacial layers in emulsions. When mixed emulsifiers are applied before homogenization, the emulsifier that adsorbs more rapidly to droplet surfaces will be present at first. However, this may be displaced during storage owing to competing adsorption with other components in the system. Globular proteins may be more difficult to dislodge when the contact has been aged or heated since denaturation causes more interfacial protein cross-linking. Proteins often have substantially higher adsorption energy per molecule than surfactants (Tween) and fatty acid diglycerol esters and hence tend to saturate fluid surfaces at much lower concentrations than surfactants. More structural insights, such as the predominance of the random coil and globular proteins and their structural stability, are needed to better understand microalgae proteins’ adsorption behavior at fluid interfaces [47].

Simões et al. [48] produced vegan emulsions containing grass pea sweet Miso regarding the rheological parameters, specifically the mechanical spectrum of emulsions. From the rheology perspective, the five formulations with 5–15% (*w*/*w*) of Miso showed the same behavior found in the present work, presenting G′ values higher than those of G″, with a slight dependence on frequency.

The shape of the curves for the emulsions with powdered aquafaba, green pea, and lupin protein formulated in the work of Cabrita et al. [49] was all similar, showing G′ higher than G″ also indicating a predominantly elastic behavior. Microalgae proteins have been studied more and more recently to stabilize fluid interfaces mostly because coalescence, flocculation, Ostwald ripening, and drainage are all reduced in emulsions and foams with high interfacial viscoelasticity [46]. Various microalgae species and fractions have shown that emulsions and foams can be stabilized, and whole disrupted cells can be used to create emulsions and foams without further fractionation or purification [46,50]. Using crude extracts is already considered a promising strategy to utilize the entire microalgae biomass with the least loss and energy input. The present work demonstrated that the residue from the extraction of highly valuable pigment from the *Spirulina* biomass could also be used to manufacture emulsions, being a practical application of the circular economy and sustainability.

From Figure 2, the results show that all formulated emulsions have similar viscoelastic behavior in the experimental frequency range, regardless of the RB/CPP ratio used as emulsifiers. The analysis of Figure 2 also shows that the emulsions with higher ratios of RB (R75CPP25, and R100CPP0) have the highest G′ and G″. When comparing the mechanical spectra of all emulsions with the standard (R0CPP100), considering the other samples, it is possible to notice that the emulsions with lower ratios of RB (R25CPP75, and R50CPP50) are more similar to the standard behavior of the R0CPP100 emulsion.

The elastic modulus G′ at 1 Hz (Figure 3) for R0CPP100, R25CPP75, and R50CPP50 showed no statistical differences (*p* > 0.05, Tukey test) between them but was higher when we increased the concentration of RB for samples R75CPP25 and R100CPP0. The same behavior was observed for viscous modulus G″ and the Plateau modulus. There is a tendency for the linear viscoelastic functions to increase with increasing concentration of RB. Emulsions with higher RS contents have G′ and G″ values characteristic of emulsions with a high degree of structuring [49].

Although the texture results revealed that the chickpea-only emulsion had higher values of the texture parameters, compared with the binary protein mixtures, in terms of linear viscoelasticity, this sample does not reveal such a high degree of structuring (low values of G′ and G″). Thus, the antagonistic effect between chickpea protein and *Spirulina* residue is not enhanced in terms of viscoelastic properties.

Moreover, considering the results from Figure 3 and comparing them with the plateau modulus obtained by Simões et al. [48] for vegan mayonnaise (3.98 ± 0.18 × 10^2^ Pa), a similar range of values was found. Raymundo et al. [51] manufactured emulsions containing pea protein and *Chlorella vulgaris,* and for the emulsion systems with the addition of microalgal biomass, the G^0^_N_ was significantly higher than for the standard. On the other hand, Batista et al. [52] studied the implications of pigment addition on emulsions’ rheological behavior and microstructure. They indicated that the phycocyanin emulsion had substantially (*p* < 0.05) higher values for the viscoelastic functions (G′, G″, and G^0^_N_) than the control, suggesting a more developed three-dimensional structure. Still, the lutein-containing emulsion had significantly (*p* < 0.05, Tukey test) lower values. The emulsion containing both pigments behaved in the middle, similar to the control emulsion.

By analyzing Figure 4, all the emulsions showed the same flow trend, as already mentioned. From the viscosity curves and η_0_ values, the behavior was even more distinct since all the concentrations of RB lead to significantly different values among the formulations ranging from 2.89 × 10^3^ (for R25CPP75) to 1.13 × 10^4^ Pa.s (R75CPP25), in addition, R25CPP75 was statistically equal to the formulation without RB (R0CPP100). Furthermore, the other Williamson parameters, namely k and m, did not change prominently for emulsions.

Comparing the values of Williamson parameters from the present work with other emulsions using non-animal proteins produced by Cabrita et al. [49] that formulated pea (PPE), aquafaba powdered (APE), lupin (LPE) and fava bean protein (FBPE) emulsions with 1.5% (*w*/*w*) protein, the lowest values presented by the authors were related to LPE emulsion (15.9 μm) that is similar to the obtained for the R75CPP25 emulsion (15.25 µm). Considering the other emulsions, all the values are higher than those obtained in the present work ranging from 34.5 to 44.2 µm.

Droplet size (d_4/3_) of Pickering emulsions stabilized by *Spirulina* protein (SPP) nanoparticles were evaluated by Guo et al. [19], and the results ranged from 18.46 to 45.52 µm, and the values depended on the concentration of nanoparticle concentration (wt%) added. The values are also similar to those found in the present work.

It is possible to observe a similar shape of the DSD curves (Figure 5), with unimodal curves, except for the emulsion containing only RB as a protein source (R100CPP0) (Figure 5). Moreover, the microscopy results show similarities in the DSD of the emulsion. This microstructural examination confirmed that all emulsions comprised distributed spherical oil droplets in an aqueous media. The droplets are closely packed, most likely due to the high oil content (>60%).

All the emulsions tend to a monomodal peak, which was also obtained by evaluating the commercial mayonnaise performed by Cabrita et al. [49]. The exception is the RB100CPP0 emulsion which shows three peaks. Regarding droplet size distribution, as already demonstrated by other authors [49,50,51,52], the processing variables, especially the changes in the protein type and/or proportions, affect the emulsion’s properties, such as texture and droplet size. This fact can explain the differences among the formulations observed in Figure 5.

These results showed that unlike the other four formulations produced, RB100CPP0 demonstrated a bimodal distribution. Such distribution shows poorer stability than unimodal ones, indicating that a combination of RB with another protein source is necessary to improve the emulsion’s stability. Further observing the DSD curve (Figure 5), the formulations containing RB ranging from 25 to 75% of the total protein added to the emulsion showed smaller droplet sizes than the emulsion without RB, once again indicating that the combination among RB and chickpea improves the stability in terms of DSD.

The structure of microalgae protein fractions is currently largely unclear. Phycobiliproteins are an exception, having been used for decades as bio-based pigments in industry and fluorescent markers in research. However, the proteins derived from microalgae are a composite of several fractions [31]. More structural insights are necessary to better understand the adsorption behavior of microalgae proteins at fluid interfaces. The predominance of random coil and globular proteins, as well as their structural stability, would be particularly useful in understanding their degree of unfolding at fluid interfaces. The interfacial structure of adsorbed microalgae protein layers is similar [53].

Based on emulsion droplet size, a few studies evaluated the surface coverage of adsorbed protein layers. Protein isolates from several microalgae species have been found to have values ranging from 0.4 to 3.3 mg/m^2^, which is in the range of protein monolayers with varying densities. The 5 mg/m^2^ was reported for crude and soluble extracts, indicating the production of multilayers and biopolymer aggregates. In the future, interfacial reflectivity or film displacement techniques will be necessary to acquire more quantitative insights into layer thickness and the presence of protein-protein or protein-polysaccharide complexes.

Even at low concentrations, the protein from *Spirulina* residual biomass can effectively stabilize emulsions, allowing for a minimal processing approach. The current study is pertinent to the food sector, looking for good emulsifying agents at low concentrations that can resist the processes utilized in food formulations.

The smallest particle diameter was presented by Ebert et al. [54]; the authors formulated emulsions containing *Chlorella sorokiniana*, prepared by high-pressure homogenization. The results showed that a concentration of 1.0% of soluble protein from *Chlorella sorokiniana* was adequate to create an emulsion with a monomodal droplet size distribution and a small volume (d_4,3_ = 232.22 nm, *n* = 10).

Concerning the emulsion colour parameters, the sensory acceptance of consumers is highly attached to the appearance of food products, and colour is one of the most critical attributes to be considered [26]. Therefore, the colour stability of the emulsions after 30 days is very expressive since no additives or colour stabilizing were added to the product. Compared with the study of emulsions containing *Spirulina*, *Chlorella vulgaris*, and *Dunaliella salina* as ingredients of low-fat emulsions that showed a significant change in color after 15 days of storage [26], the formulations containing RB were more stable considering the color preservation.

Added to the color evaluation, according to the literature, the antioxidant activity is *Spirulina’s* most highlighted biological effect, and fortunately, the present work shows that the RB also possesses this ability. Bermejo-Bescós et al. [55] evaluated *Spirulina’s* antioxidant activity in vitro and found their capacity to inhibit hydroxyl radical-mediated deoxyribose degradation and peroxyl radical production. Moreover, Abdelkhalek et al. [56] assumed that *Spirulina* exerts a protective effect by indirectly leading to the improvement of endogenous enzymatic antioxidants and/or directly scavenging free radicals preventing lipid peroxidation in vivo. Thus, using RB as a food ingredient should prevent oxidative stress due to the interaction with various ROS (i.e., hydroxyl radical (•OH)) and lipid peroxidation, enhancing the endogenous enzymatic antioxidant profile in vivo.

Therefore, besides successfully incorporating RB as a protein source to be applied in emulsions, the antioxidant potential as a biological effect of the residue was also an important feature to be highlighted in the present work. In addition, with the high concentration of proteins found in the residual biomass from *Spirulina*, efforts must be made to escalate biorefinery, making microalga protein sources an alternative for stabilizing emulsions.

## 4. Materials and Methods

### 4.1. Spirulina Residual Biomass (RB)

*Spirulina* used in the present work was provided by Fazenda Tamanduá^®^ (Santa Teresinha, Paraíba, Brazil). *Arthrospira platensis* is cultivated in the pure waters of the subsoil of Fazenda Tamanduá^®^, guaranteeing the absence of contamination by pesticides or heavy metals, presenting a biodynamic and organic certification. To start the present work, *Spirulina* biomass was thawed 1 h before the extraction and was processed in an analytical knife mill (IKA^®^ A11 Basic, Königswinter, Germany) to separate the particles through a 0.106 mm mesh/Tyler [30,57]. Distilled water was added to the sample, kept at room temperature, and protected from light for 1 h. The mixture was centrifuged for 15 min at 10,000 rpm in 4 cycles (NT 816, Nova Analítica^®^, Diadema, Brazil), and the supernatant was separated from the RB. Then, with the liquid fraction of the extract (C-PC), the samples were filtered using vacuum filtration and through a 0.22 μm hydrophilic syringe filter and kept in an ultra freezer at −40 °C [30]. The RB was also frozen, and before its utilization as an ingredient, the material was lyophilized. After that, it was processed in an analytical knife mill (IKA^®^ A11 Basic, Königswinter, Germany) to separate the particles through a 0.106 mm mesh/Tyler, ensuring a homogeneous particle size distribution. Since water was the only solvent utilized during C-PC extraction, the RB is safe to be used as a food ingredient.

### 4.2. Emulsions Formulation

Oil-in-water emulsions in batches of 100 g were manufactured. Before the emulsion preparation, the vegetable proteins were hydrated in distilled water for 30 min with magnetic stirring at room temperature. Emulsions were stabilized with 3.0% (*w*/*w*) total protein. The total protein percentual was composed of *Spirulina* RB and chickpea protein isolate (70%). Five formulations were prepared (in triplicate each) as follows, R100CPP0 (100% chickpea protein, without *Spirulina* RB), R25CPP75 (25% of *Spirulina* RB and 75% chickpea protein), R50CPP50 (50% *Spirulina* RB and 50% chickpea protein), R75CPP25 (75% *Spirulina* RB and 25% chickpea protein) and R100CPP0 (100% *Spirulina* RB without chickpea protein).

According to Cabrita et al. [46], once the protein was hydrated, sunflower oil (65% *w*/*w*) was added under agitation in an Ultra Turrax T- 25 (IKA^®^, Königswinter, Germany) homogenizer at 14,000 rpm for 10 min. The emulsions were stowed in cylindrical glass jars (62 mm diameter, 56 mm height) and chilled (4 °C) for 24 h to reach equilibrium before measurements.

### 4.3. Rheology Analysis and Measurements

Linear viscoelasticity and steady-state flow measurements were performed using a Haake MARS III (Thermo Fisher Scientific, Waltham, MA, USA) outfitted with a UTC Peltier. Small amplitude oscillatory shear (SAOS) measurements were acquired with a cone-and-plate sensor system (35 mm, 2°) in the linear viscoelastic region, which had previously been assessed for each sample to obtain the mechanical spectrum (registering G′ and G″ as a function of frequency) and the loss tangent (tan δ = G″/G′). The plateau modulus (G^0^_N_) was calculated as the value of G′ obtained for the smallest loss tangent (G″/G′) [58]. The experiment was repeated five times.

Steady-state flow waveforms were produced by varying the shear rate from 10^−8^ to 500 s^−1^. To avoid the slip effect, a serrated parallel plate system (P20-20 mm diameter) was employed for these experiments [59]. The experiment was repeated five times.

The viscosity versus shear rate curve was adjusted to the Williamson model (1) according to Álvarez-Castillo et al. [37], using the Origin 2023 software (Origin Pro 2023, OriginLab^®^).

(1) $$\eta =\frac{\eta_{0}}{1+{\left(k\dot{\gamma }\right)}^{m}}$$

where η_0_ = the zero shear rate limiting viscosity at low shear rates (expressed in Pa for s), *k* = consistency coefficient (expressed in Pa for s), and m = shear-thinning index (dimensionless).

### 4.4. Texture Analysis and Measurements

Texture Profile Analysis (TPA) was performed using a texturometer TAXT plus texturometer (Stable Micro-Systems^®^, Surrey, UK) with a 5 kg load cell; the test was repeated nine times at 20 °C in a temperature-controlled environment for each sample. A Perspex cylindrical probe with a 19 mm diameter and a depth of 15 mm was used to enter the samples. Firmness (N) and adhesiveness (N.s^−1^) were calculated using the force versus time texturograms [49].

### 4.5. Evaluation of Emulsion Stability and Structure

At 20 °C, the droplet diameter distribution of the emulsions was determined using a laser diffraction device (Mastersizer 2000; Malvern Instruments, Malvern, UK). The diameter *d*_4,3_ (2) for each formulation was calculated using the droplet size distributions [48]. The experiment was repeated four times.

(2) $$d_{4,3}=\frac{\sum n_{i}d_{i}{}^{4}}{\sum n_{i}d_{i}{}^{3}}$$

### 4.6. Antioxidant Activity

To extract the antioxidant compounds, samples were immersed in methanol (1:10 *w*/*v*), stirred for 60 min at room temperature, and centrifuged at 10,000 rpm at 20 °C for 10 min (NT 816, Nova Analítica^®^, Brazil) [60]. Hence, the supernatant was used as the antioxidant extract for the analysis using DPPH [61] and FRAP [62] methods. For both, a calibration curve was done using Trolox, and the results were expressed in Trolox equivalents per 100 g.

All the solutions described by the methodology were prepared for both antioxidant activity methods. For DPPH, a 2,2-diphenyl-1-picrylhydrazyl (0.10 mM) solution was prepared to conduct the analysis, and for the FRAP method, 2,4,6-Tri(2-pyridyl)-1,3,5-triazine (10 mM) solution was prepared and distilled water served as a blank. Water was utilized as a negative control instead of extracts. The calibration curve parameters derived from its linear regression were used to determine the Trolox equivalent values from the absorbance measurements [49]. The analysis was repeated four times.

### 4.7. Microscopy

The microstructure of the emulsions was evaluated using visible light microscopy using a microscope (Leitz-Dialux Leica 20, Famalicão, Portugal). An aliquot of 10 µL of the samples was immediately put on a transparent slide (76 × 26 mm), gently covered by coverslips (18 × 18 mm). An optical microscope Leica SP2 (Munich, Germany) with a 4 MP digital camera was used for these investigations. Warm visible light was delivered through the samples while a 10-magnification (10×) objective was utilized.

### 4.8. Color Measurements

The color of the emulsions was tested twice, at t = 0 (as soon as the emulsions were made) and t = 30 (30 days after the preparation), using a Minolta CR-400 (Tokyo, Japan) colorimeter with standard illuminant D65 at a visual angle of 2°. Data were expressed according to the CIELab system (L*, a*, b*), described by the International Commission of Illustration. All samples were measured, in triplicate (each replica four times), under the same light conditions, at room temperature, using a standard white tile (L* = 86.70, a* = 0.32, b* = 0.34). The Hue angle (h), which indicates the color angle, was calculated by Equation (3). Results were analyzed in color space, and differences in L* (luminosity), a* (from greenness to redness), and b* (from blueness to yellowness) indicate the low (−) to high (+) value of color parameters. The color difference (ΔE, Equations (4)–(7)) was also calculated from the initial (t = 0) and final (t = 30) values of L*, a*, and b* of each formulation, relative to the control were also measured, as well as the total color difference from the control formulation.

(3) $${h\;({}^{\circ})=180+\tan}^{-1}\left(\frac{b^{*}}{a^{*}}\right)\mathrm{when}\;(-a^{*},\;+b^{*})\;\mathrm{or}\;(-a^{*},\;-b^{*})$$

ΔE * = [(ΔL*)^2^ + (Δa*)^2^ + (Δb*)^2^]^1/2^ (4)

(5) $${\Delta L}^{*}{=L}_{0}^{*}-{\;L}_{t}^{*}$$

(6) $${\Delta a}^{*}{=a}_{0}^{*}-{\;a}_{t}^{*}$$

(7) $${\Delta b}^{*}{=b}_{0}^{*}-{\;b}_{t}^{*}$$

### 4.9. Statistical Analysis

The samples were prepared in triplicate; the measures were done at least three times, as detailed in each methodology section. Statistica Software (version 7.0) was used to conduct the analysis of variance (ANOVA), using Tukey as the *post hoc* test to compare three or more samples with a 95% degree of confidence (*p* = 0.05).

## 5. Conclusions

The results showed that it was possible to formulate stable protein-rich emulsions using recovering matter rich in protein from *Spirulina* biomass as an innovative food ingredient. All the concentrations used of the Residual Biomass promoted the formulation of emulsions presenting interesting rheological parameters compared with a more traditional protein source such as chickpea. The emulsions were also a source of antioxidant compounds and maintained the color after 30 days of production. Overall, all emulsions presented volumetric diameters (d_4,3_) ranging from 15.25 to 33.04 µm, and the antioxidant activity, considering both methods, was very expressive, proofing that the residual biomass, besides its rheological and textural excellent properties, also presents the potential of a functional ingredient.

The present work showed that it is possible to apply a biorefinery approach allowing the incorporation of an innovative clean-label ingredient that can be used in food products as a protein source to increase health benefits such as antioxidant activity, appealing to consumers searching for bold color products aligned with the sustainability and circular economy concepts.

## Figures and Tables

**Table 1:** The flow curve parameters (zero shear rates limiting viscosity, η_0_, consistency coefficient, k, deformation thinning rate, m) according to the Williamson’s model adjustment, and the respective R^2^ values of the different emulsions with 3% (*w*/*w*) of different combinations of proteins (chickpea protein and *Spirulina* residual biomass—RB).

Sample	η_0_ (10^3^ Pa.s)	k (Pa.s)	m (Dimensionless)
R0CPP100	5.61 ^d,b^ ± 0.62	282.0 ^c^ ± 26.5	1.49 ^a^ ± 0.1
R25CPP75	2.89 ^c^ ± 0.29	210.7 ^b^ ± 25.8	1.12 ^a^ ± 0.3
R50CPP50	4.46 ^b,c,d^ ± 0.61	270.0 ^b,c^ ± 26.1	1.42 ^a^ ± 0.2
R75CPP25	6.13 ^b^ ± 0.32	252.7 ^b,c^ ± 25.2	1.25 ^a^ ± 0.2
R100CPP0	8.89 ^a^ ± 1.30	500.0 ^a^ ± 1.00	1.34 ^a^ ± 0.1

Values are shown in mean ± standard deviation. According to the Tukey test, different letters in the same column indicate significantly different results (*p* < 0.05). R0CPP100 (100% chickpea), R25CPP75 (25% RB and 75% chickpea), R50CPP50 (50% RB and 50% chickpea), R75CPP25 (75% RB and 25% chickpea) and R100CPP0 (100% RB without chickpea).

**Table 2:** Color parameters of emulsions formulations (initial and final times) for brightness (L*), amount of red or green (a*), amount of yellow or blue (b*), and color (Hue angle).

Sample	t = 0				t = 30 Days			
	L*	a*	b*	Hue Angle	L*	a*	b*	Hue Angle
R0CPP100	85.46 ^a^ ± 1.25	−0.63 ^a^ ± 0.01	11.14 ^a^ ± 0.85	93.7 ^a^ ± 0.23	86.44 ^a^ ± 0.02	−0.63 ^a^ ± 0.00	12.26 ^a^ ± 0.13	92.96 ^a^ ± 0.03
R25CPP75	55.21 ^a^ ± 0.51	−9.10 ^a^ ± 0.10	13.80 ^a^ ± 0.07	123.41 ^a^ ± 0.28	54.41 ^a^ ± 0.53	−9.18 ^a^ ± 0.07	13.65 ^a^ ± 0.02	123.93 ^a^ ± 0.18
R50CPP50	47.44 ^a^ ± 0.32	−9.20 ^a^ ± 0.06	12.46 ^a^ ± 0.17	126.45 ^a^ ± 0.27	46.55 ^a^ ± 0.27	−9.27 ^a^ ± 0.14	12.44 ^a^ ± 0.00	126.67 ^a^ ± 0.41
R75CPP25	43.66 ^a^ ± 0.24	−9.49 ^a^ ± 0.15	11.59 ^a^ ± 0.34	129.33 ^a^ ± 0.68	43.53 ^a^ ± 0.14	−9.57 ^a^ ± 0.07	11.48 ^a^ ± 0.07	129.80 ^a^ ± 0.30
R100CPP0	41.16 ^a^ ± 0.41	−9.52 ^a^ ± 0.09	11.51 ^a^ ± 0.19	132.18 ^a^ ± 0.30	40.64 ^a^ ± 0.58	−9.55 ^a^ ± 0.06	10.68 ^a^ ± 0.05	131.82 ^a^ ± 0.06

Values are shown in mean ± standard deviation. According to the Tukey test, different letters in the same line indicate significantly different results (*p* < 0.05), considering each parameter evaluated. R0CPP100 (100% chickpea), R25CPP75 (25% RB and 75% chickpea), R50CPP50 (50% RB and 50% chickpea), R75CPP25 (75% RB and 25% chickpea) and R100CPP0 (100% RB without chickpea).

**Table 3:** Antioxidant activity of the emulsions with 3% (*w*/*w*) of different combinations of proteins (chickpea protein and RB), determined by DPPH and FRAP methods.

Samples	Antioxidant Activity (µmol Trolox/100 g)	
	DPPH	FRAP
R0CPP100	15.4 ^c^ ± 0.16	169.4 ^c^ ± 5.75
R25CPP75	16.6 ^c^ ± 2.17	181.7 ^c^ ± 3.34
R50CPP50	26.2 ^b^ ± 2.37	226.6 ^b^ ± 10.69
R75CPP25	29.6 ^b^ ± 3.47	249.0 ^b^ ± 3.08
R100CPP0	43.0 ^a^ ± 1.50	343.9 ^a^ ± 21.12

Values are shown in mean ± standard deviation. Equal letters indicate no significant difference between the means (*t*-test, *p* > 0.05) in the same column. R0CPP100 (100% chickpea), R25CPP75 (25% RB and 75% chickpea), R50CPP50 (50% RB and 50% chickpea), R75CPP25 (75% RB and 25% chickpea) and R100CPP0 (100% RB without chickpea).

## Data Availability

The data supporting reported results can be accessed via email for the author (anna.braga@unifesp.br).

## Sample Availability

Samples of the compounds are available from the authors.
